# Application of individual brain connectome in chronic ischemia: mapping symptoms before and after reperfusion

**DOI:** 10.1002/mco2.585

**Published:** 2024-06-02

**Authors:** Yu Lei, Xin Zhang, Wei Ni, Chao Gao, Yanjiang Li, Heng Yang, Xinjie Gao, Ding Xia, Xia Zhang, Karol Osipowicz, Stephane Doyen, Michael E. Sughrue, Yuxiang Gu, Ying Mao

**Affiliations:** ^1^ Department of Neurosurgery Huashan Hospital Fudan University Shanghai China; ^2^ National Center for Neurological Disorders Shanghai China; ^3^ Shanghai Key Laboratory of Brain Function and Restoration and Neural Regeneration Shanghai China; ^4^ Neurosurgical Institute of Fudan University Shanghai China; ^5^ Shanghai Clinical Medical Center of Neurosurgery Shanghai China; ^6^ Department of Radiology Huashan Hospital Fudan University Shanghai China; ^7^ International Joint Research Center on Precision Brain Medicine XD Group Hospital Xi'an China; ^8^ Omniscient Neurotechnology Sydney New South Wales Australia

**Keywords:** brain ischemia, cerebral revascularization, machine learning, neural networks

## Abstract

How brain functions in the distorted ischemic state before and after reperfusion is unclear. It is also uncertain whether there are any indicators within ischemic brain that could predict surgical outcomes. To alleviate these issues, we applied individual brain connectome in chronic steno‐occlusive vasculopathy (CSOV) to map both ischemic symptoms and their postbypass changes. A total of 499 bypasses in 455 CSOV patients were collected and followed up for 47.8 ± 20.5 months. Using multimodal parcellation with connectivity‐based and pathological distortion‐independent approach, areal MR features of brain connectome were generated with three measurements of functional connectivity (FC), structural connectivity, and PageRank centrality at the single‐subject level. Thirty‐three machine‐learning models were then trained with clinical and areal MR features to obtain acceptable classifiers for both ischemic symptoms and their postbypass changes, among which, 11 were deemed acceptable (AUC > 0.7). Notably, the FC feature‐based model for long‐term neurological outcomes performed very well (AUC > 0.8). Finally, a Shapley additive explanations plot was adopted to extract important individual features in acceptable models to generate “fingerprints” of brain connectome. This study not only establishes brain connectomic fingerprint databases for brain ischemia with distortion, but also provides informative insights for how brain functions before and after reperfusion.

## Graphical abstract

We applied individual brain connectome in chronic steno‐occlusive vasculopathy to map both ischemic symptoms and their postbypass changes. Using multimodal parcellation with connectivity‐based and pathological distortion‐independent approach, areal MR features of brain connectome were generated with three measurements at the single‐subject level. Machine‐learning models were then trained with clinical and areal MR features to obtain acceptable classifiers for both ischemic symptoms and their postbypass changes. Finally, a Shapley additive explanations plot was adopted to extract important individual features in acceptable models to generate “fingerprints” of brain connectome.

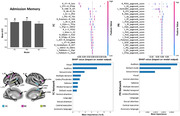

## 1 INTRODUCTION

Bypass surgery is effective for the treatment of chronic steno‐occlusive vasculopathy (CSOV) by reducing the risk of future stroke, reducing frequency of transient ischemic attacks, and improving long‐term cognitive function and activities of daily living.^1^, ^2^, ^3^ The surgery is recommended to be performed as soon as it can be reasonably scheduled after the acute phase.^4^, ^5^ Nevertheless, not all patients benefit from this gold‐standard surgery due to postoperative ischemia, hyperperfusion syndrome (HPS) and other unknown reasons.^6^, ^7^ Regarding these high‐risk patients, surgical indication should be more strict and perioperative management should be more enforced. However, it is difficult to discriminate high‐risk patients from the whole CSOV group prior to surgery. What is worse, in absence of effective approaches, mapping neurologic and psychiatric symptoms in CSOV is still a traditional challenge.

Some clinical factors have been proposed as predictors of postbypass complications sporadically, such as the advanced age, left‐side surgery, some angioarchitectural, and hemodynamic features.^5^, ^8^, ^9^ However, these factors are determined at group level and thus cannot be prognostic to the individual patient. The promising Berlin grading system is proposed to predict surgical outcomes based on individual ischemic status, but one involved factor of cerebrovascular reserve capacity cannot be performed in some countries, which limits the application of this system.^10^ Thus, novel measurements of brain ischemia should be developed to evaluate clinical symptoms and to predict surgical outcomes.

The complicated pathogenesis of the CSOV leads to abnormal brain activation and thereafter observable clinical symptoms. Clinically, lesions in different brain regions often cause similar symptoms, which is explained by the theory that symptoms correspond more closely to networks of connected regions rather than specific regions.^11^ Therefore, features of brain connectome are valuable, individualized characteristics that reflect brain abnormalities caused by disease.^12^ The promising approach of symptom mapping has been applied to many neuropsychiatric symptoms, but is rarely used in brain ischemia due to its common pathological distortion. Neuroscientists find it difficult to parcellate distort cortex at the single‐subject level or make an accurate identification of areal boundary.

Fortunately, a novel connectivity‐based parcellation approach has been proposed to acquire single‐subject atlas independent of brain pathological distortion and can be used to solve the above problems.^13^, ^14^ After extracting individual areal features of the brain connectome in patients with CSOV using this approach, we trained machine‐learning (ML) models to recognize clinical neurological, and cognitive status at admission, long‐term follow‐ups, as well as postoperative aggravated or newly onset complications. Finally, important areal features in acceptable models were generated as individual fingerprints via a Shapley additive explanations (SHAP) plot. This study not only establishes the first brain connectomic fingerprint database for brain ischemia with distortion, but also provides informative insights for how brain functions after reperfusion.

## 2 RESULTS

### 2.1 Clinical features

Four hundred fifty‐five patients of CSOV with 499 bypasses were identified from May 2013 to 2021 (Table 1). Postoperative ischemic stroke and HPS were recorded in 31 (6.2%) and 166 (33.3%) bypasses, respectively. All involved bypasses were confirmed patency through angiography at 6‐month follow‐up. The mean follow‐up was 47.8 ± 20.5 months. Twelve (2.4%) procedures failed to receive neuropsychological follow‐up due to severe neurological deficits.

**TABLE 1:** Clinical characteristics of hemispheres with CSOV.

	Total (*n* = 499)	General campus (*n* = 142)	North campus (*n* = 301)	West campus (*n* = 56)
Age (years)	42.7 ± 11.4	41.6 ± 10.5	43.0 ± 11.7	43.7 ± 12.4
Male (%)	227 (45.5)	65 (45.8)	139 (46.2)	23 (41.1)
Left side (%)	254 (50.9)	76 (53.5)	148 (49.2)	30 (53.6)
Ischemic type (%)	378 (75.8)	99 (70.0)	236 (78.4)	43 (76.8)
Past medical history (%)				
Hypertension	114 (22.8)	30 (21.1)	70 (23.3)	14 (25.0)
Diabetes	102 (20.4)	25 (17.6)	67 (22.3)	10 (17.9)
Hyperlipidemia	86 (17.2)	24 (16.9)	53 (17.6)	9 (16.1)
Current Smoking	100 (20.0)	27 (19.0)	61 (20.3)	12 (21.4)
Clinical symptoms at admission				
NIHSS score	1.8 ± 2.1	1.8 ± 2.0	1.7 ± 2.0	1.8 ± 2.5
MMSE score	25.0 ± 5.0	25.2 ± 4.8	25.1 ± 4.9	23.9 ± 6.2
MES score	73.5 ± 21.0	69.8 ± 22.6	75.5 ± 19.5	72.0 ± 23.4
Perioperative surgical outcomes				
NIHSS score	3.3 ± 4.5	3.0 ± 4.4	3.4 ± 4.6	3.9 ± 4.0
mRS score	1.9 ± 1.5	1.8 ± 1.5	1.9 ± 1.6	2.2 ± 1.5
Aphasia (%)^a^	102 (20.4)	24 (16.9)	66 (21.9)	12 (21.4)
Motor paresis (%)^a^	119 (23.8)	28 (19.7)	76 (25.2)	15 (26.8)
Long‐term surgical outcomes				
NIHSS score	1.4 ± 4.1	1.4 ± 4.3	1.5 ± 4.0	1.5 ± 3.7
mRS score	0.7 ± 1.2	0.6 ± 1.1	0.8 ± 1.2	0.8 ± 1.2
MMSE score	26.5 ± 4.6	26.6 ± 4.4	26.5 ± 4.6	25.8 ± 5.2
MES score	80.4 ± 20.0	79.3 ± 21.8	80.8 ± 19.2	81.3 ± 19.6

*Note*: a means postoperative newly onset or aggravated symptoms.

### 2.2 Eleven acceptable ML models and four effective tests are generated

A total of 33 ML models were developed for the 11 tests, among which 11 models were considered acceptable (Table 2). The very good performance is noted in a functional connectivity (FC) model to predict long‐term National Institutes of Health Stroke Scale (NIHSS) changes, while the good performance is revealed in six models for admission status recognition, two models for short‐term outcomes prediction, and two models for long‐term outcomes prediction. Afterward, three effective tests and one very effective test are generated, among which, the sensorimotor network (SMN) and default mode network (DMN) are top contributors in all good models (Table 3).

**TABLE 2:** Performances of machine‐learning models based on individual clinical features and brain connectome (shown as AUC ± standard deviation).

	Models		
Tests	Functional connectivity	Structural connectivity	PageRank centrality
Clinical symptoms localization at admission			
NIHSS score	0.688 ± 0.044	0.652 ± 0.049	0.592 ± 0.044
Memory	0.708 ± 0.048^a^	0.662 ± 0.062	0.737 ± 0.049^a^
Executive function/attention	0.719 ± 0.116^a^	0.766 ± 0.100^a^	0.764 ± 0.065^a^
Language	0.668 ± 0.064	0.688 ± 0.080	0.745 ± 0.103^a^
Visuospatial function	0.633 ± 0.080	0.644 ± 0.079	0.626 ± 0.110
Prediction of short‐term surgical recovery			
NIHSS changes	0.663 ± 0.047	0.602 ± 0.074	0.656 ± 0.039
Aphasia	0.668 ± 0.070	0.701 ± 0.066^a^	0.714 ± 0.058^a^
Motor paresis	0.659 ± 0.083	0.636 ± 0.061	0.596 ± 0.080
Prediction of long‐term surgical recovery			
NIHSS changes	0.817 ± 0.112^b^	0.733 ± 0.085^a^	0.620 ± 0.068
MMSE changes	0.640 ± 0.104	0.619 ± 0.208	0.686 ± 0.182
MES changes	0.794 ± 0.071^a^	0.633 ± 0.197	0.680 ± 0.171

^a^ AUC over 0.7 implies a good performance model.

^b^ AUC over 0.8 implies a very good performance model.

**TABLE 3:** Brain networks with important contribution in tests with at least two acceptable models.

		Brain networks	
Tests	Models	Sensor cortex systems	Task‐positive systems
Admission memory	FC, PR	Auditory network; Sensorimotor network^a^	Default mode network^a^
Admission executive function/attention	FC, SC, PR	Limbic/Paralimbic network; Sensorimotor network^a^; Visual network	Default mode network^a^; Multiple demand network; Salience network; Ventral attention network
Short‐term aphasia	SC, PR	Sensorimotor network^a^	Default mode network^a^
Long‐term NIHSS changes	FC, SC	Auditory network; language network; limbic/paralimbic network; sensorimotor network^a^; visual network	Default mode network^a^; dorsal attention network; medial temporal region; multiple demand network; salience network; ventral attention network

^a^ Networks as an important contributor in all tests.

### 2.3 Individual brain connectomic fingerprints for symptoms before reperfusion

**FIGURE 1:**
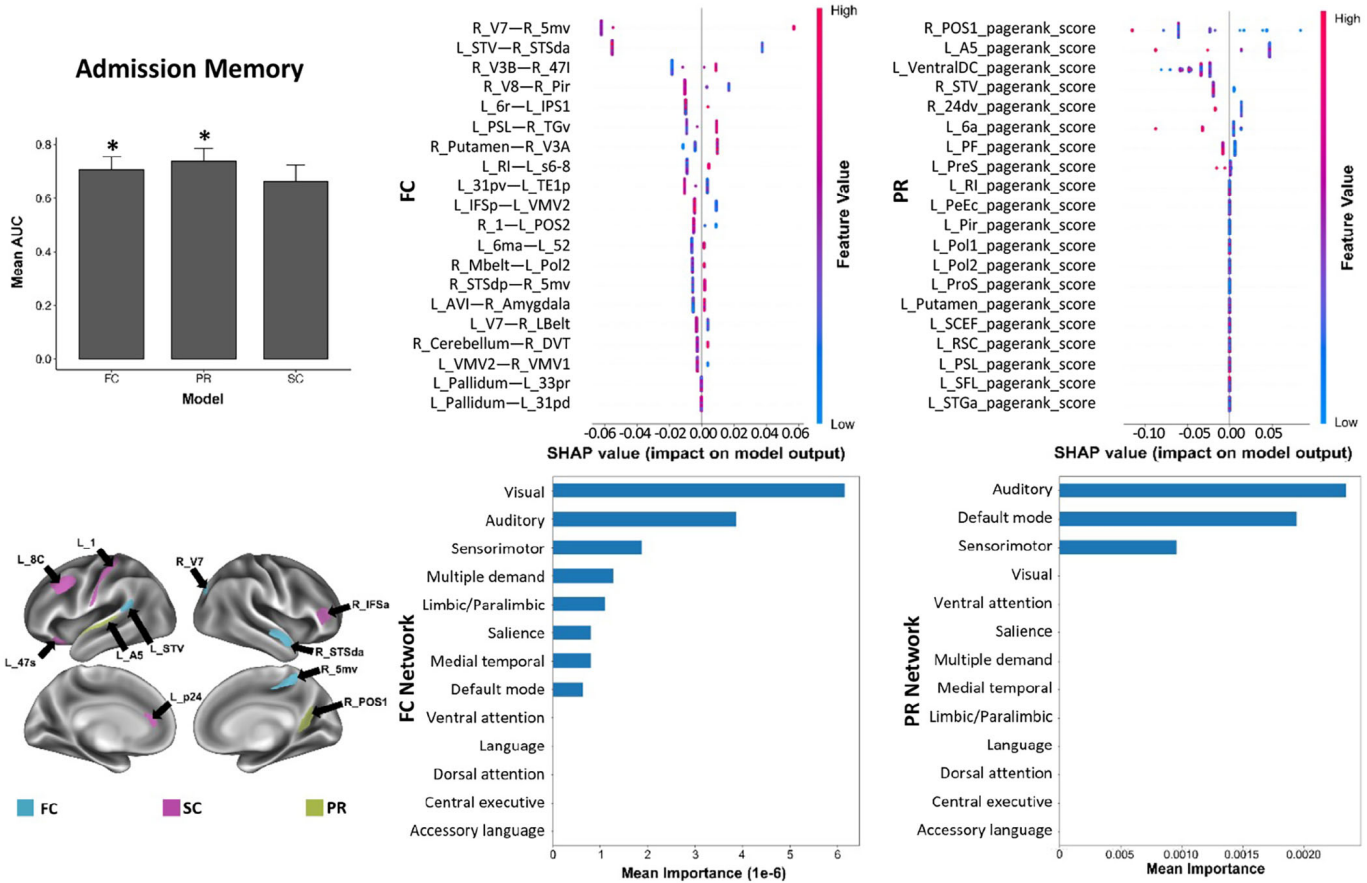
The SHAP plot of feature importance for models classifying tests of admission memory at the single‐subject level. Top areal features of brain connectome from each model are represented on a model brain. One asterisk means good performance of a model.

Performances of ML models are acceptable in recognizing memory with FC and PageRank centrality (PR) analyses, executive function/attention with FC, structural connectivity (SC), and PR analyses, as well as language with PR analysis. In SHAP analysis of memory, a high FC between the right V7 and right 5mv, and a low FC between left STV and right STSda are the top two features, while the visual network has the highest contribution at the network level (Figure 1). Besides, a low PR score of the right POS1 and left A5 contributes most, while the auditory network is the top feature at the network level. In addition, three networks are noted as important contributors in both FC and PR models (Table 3).

**FIGURE 2:**
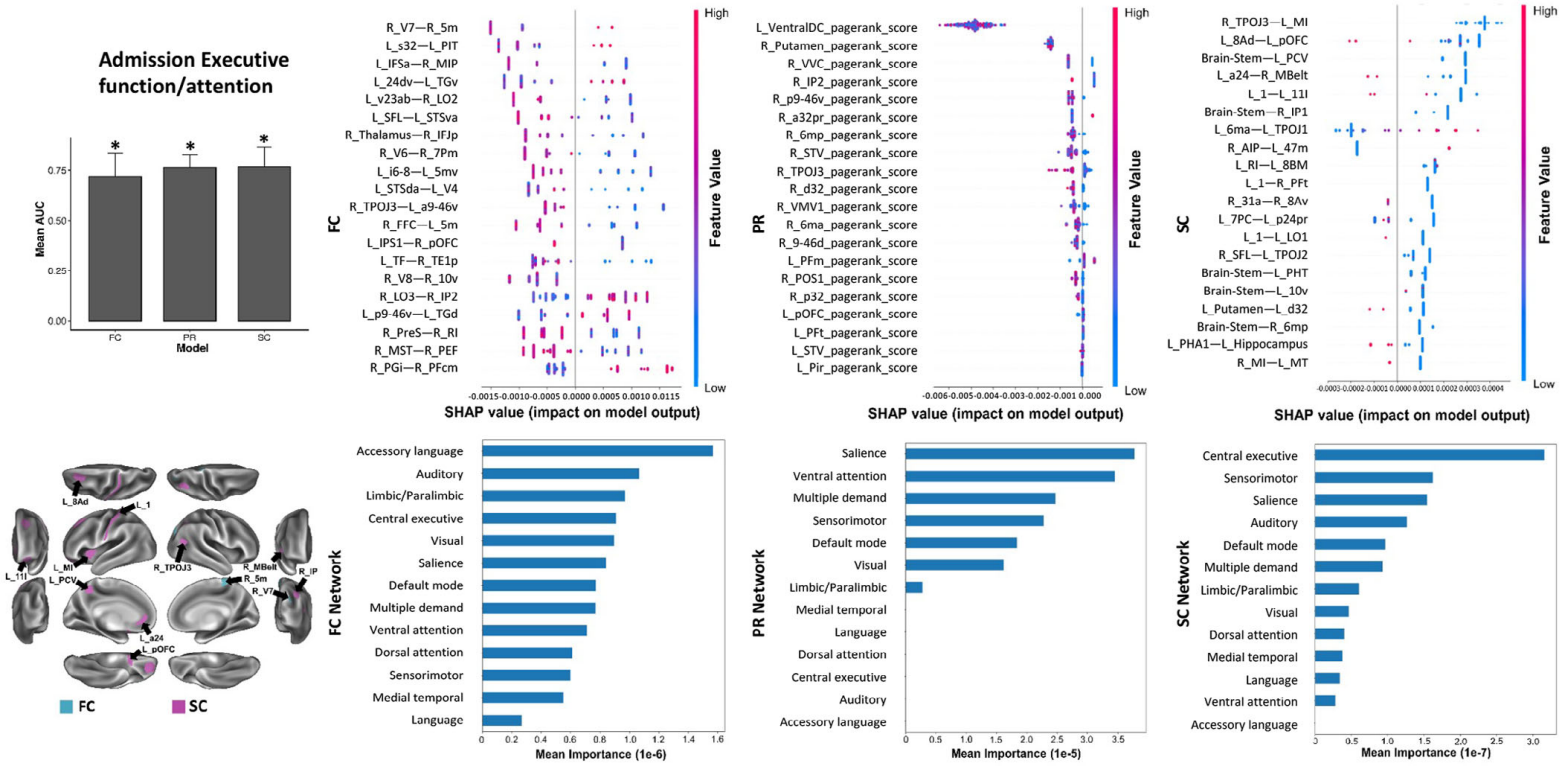
The SHAP plot of feature importance for models classifying tests of admission executive function/attention at the single‐subject level. Note that the brainstem, and left ventral diencephalon are also top features of the SC and PR models, respectively. Top areal features of brain connectome from each model are represented on a model brain. One asterisk means good performance of a model.

Referring to the executive function/attention, the FC between the right V7 and right 5m is the top feature, though there is significant overlap among features, and none particularly stands out. Among the networks, the accessory language network is the top feature (Figure 2). Next, a low SC between the right TPOJ3 and left MI is the top feature, while the central executive network contributes most at the network level. Finally, a high PR score of the left ventralDC is the top feature, and the salience network ranks the top at the network level. Notably, seven networks contribute to all three models (Table 3).

**FIGURE 3:**
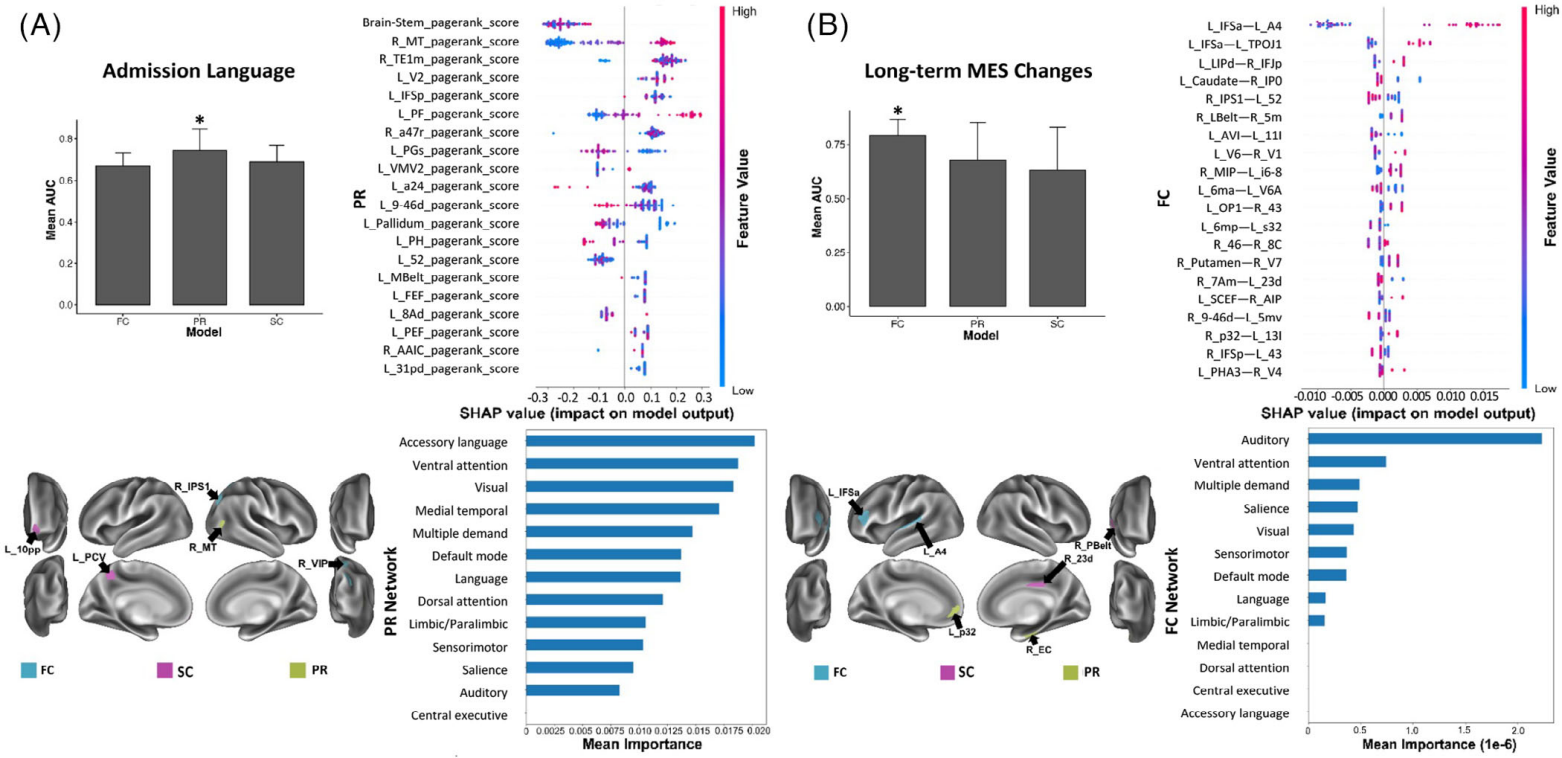
The SHAP plot of feature importance for models classifying tests of admission language (A) and long‐term MES changes (B) at the single‐subject level. Note that the brainstem is also a top feature in the PR model in testing language. Top areal features of brain connectome from each model are represented on a model brain. One asterisk means good performance of a model.

Referring to the language, a high PR of the brainstem and a low PR of the right MT are the top features, though there is a great degree of overlap in the SHAP values of features. At the network level, the accessory language network has the highest mean importance (Figure 3A).

### 2.4 Individual brain connectomic fingerprints for symptoms shortly after reperfusion

**FIGURE 4:**
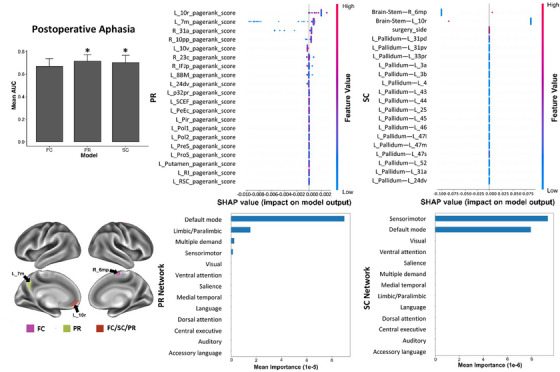
The SHAP plot of feature importance for models classifying tests of postoperative aphasia at the single‐subject level. Note that the brainstem is also a top feature in the SC model. Top areal features of brain connectome from each model are represented on a model brain. One asterisk means good performance of a model.

Performances of ML models is acceptable in predicting postoperative aphasia with SC and PR analyses. In SHAP analysis, a high SC between the brainstem and right 6mp, and a low FC between the brainstem and left 10r are top two features, while the SMN is the top feature at the network level (Figure 4). Besides, a high PR score of left 10r is the top feature, though the data show large variability in individual observations. The DMN is the top feature at the network level. In addition, two networks are found to contribute in both SC and PR models (Table 3).

### 2.5 Individual brain connectomic fingerprints for long‐term symptoms after reperfusion

**FIGURE 5:**
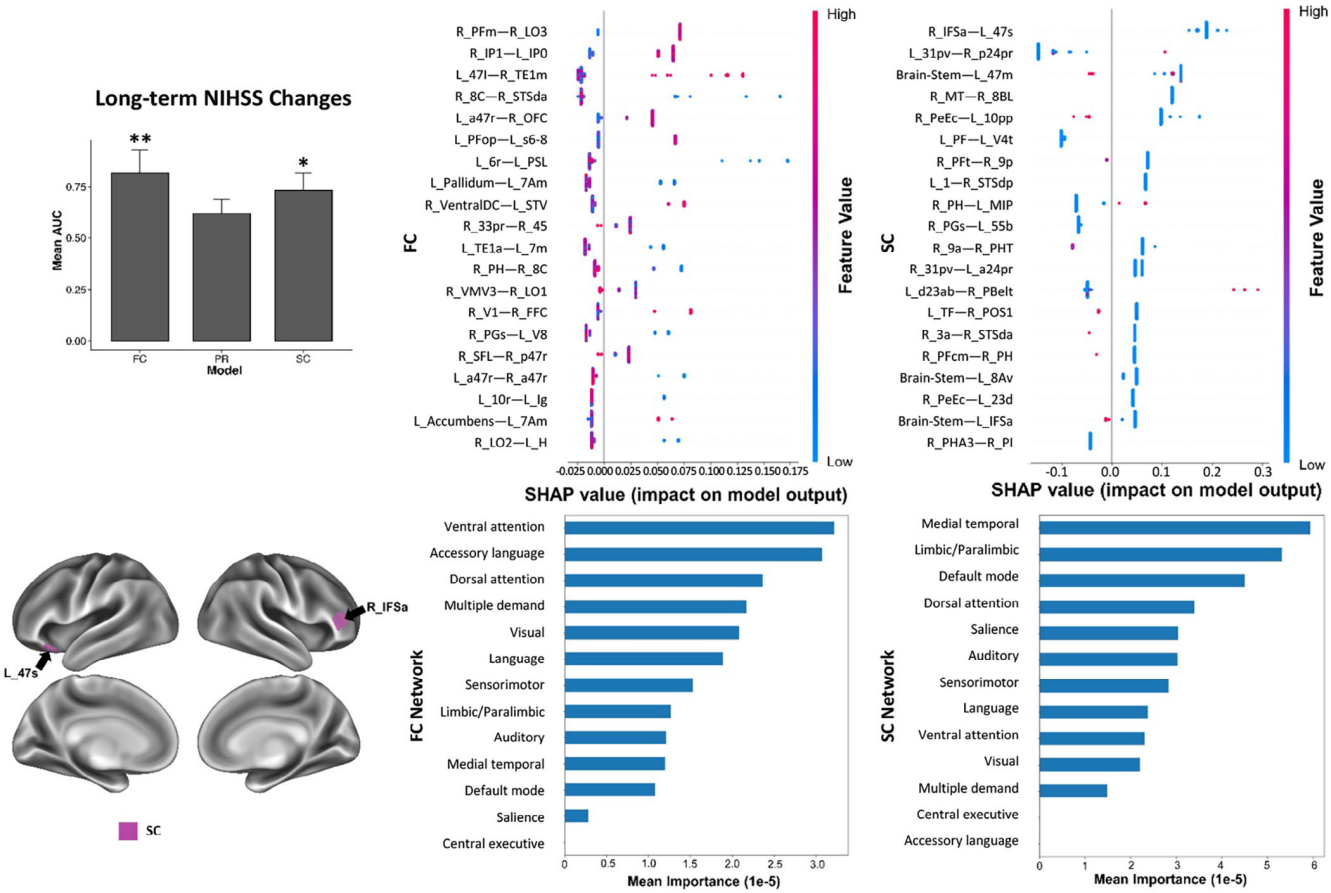
The SHAP plot of feature importance for models classifying tests of long‐term NIHSS changes at the single‐subject level. Note that the brainstem is also a top feature in the SC model. Top areal features of brain connectome from each model are represented on a model brain. One asterisk means good performance of a model. Two asterisks mean very good performance.

Performances of ML models are excellent in predicting long‐term NIHSS changes with FC analysis and acceptable with SC analysis. Besides, the performance is also acceptable in predicting long‐term Memory and Executive Screening (MES) changes with FC analysis. In SHAP analysis of NIHSS changes, there is significant overlap among FC features at the regional level and none stands out, while among the networks, the ventral attention network ranks the top (Figure 5). Furthermore, the low SC between the right IFSa and left 47s is the top feature, while the medial temporal region and the limbic/paralimbic network are the top two features at the network level. In addition, 11 networks are shown as important contributors in both FC and SC models (Table 3).

Referring to the MES changes, the high FC between left IFSa and left A4 is the top feature, while the auditory network is the top feature at the network level (Figure 3B). In addition, the ML models with poor performance (AUC < 0.7) are detailed in Supplementary Materials.

## 3 DISCUSSION

Clinical neurological and cognitive status are both reflected in the performance of brain networks and connection of their constituent parcels. Disruptions to specific networks or their components produce discrete clinical symptoms. Studies based on group‐average parcellation often overlook inaccurate alignment of regions across subjects due to anatomical distortion of diseased brain. Moreover, the nonergodicity of group effects limits the clinical utility of most researches. This study utilizes connectivity‐based parcellation at single‐subject level to acquire areal “fingerprint” of brain connectome and trains ML classifiers with samples from three independent cohorts to identify key network features that can reflect ischemic status and possibility of benefits after reperfusion. Our results demonstrate that impairment in memory, executive function/attention, and language can be recognized at admission. Additionally, postoperative newly onset or aggravated aphasia, long‐term neurological improvement, as well as improvement of memory and executive function/attention, can also be predicted.

The connectomic analyses of areal FC, SC, and PR provide network information in three aspects. The SC is extracted in terms of fiber bundles according to the regions they interconnect and stands for undirected anatomical links. The FC is calculated by correlation between nodal activities and stands for undirected statistical dependencies. In addition, the PR algorithm is developed based on link structure of websites to sort pages with importance. The distributed PR scores is commonly used to measure network topological characteristics of nodal prioritization.^15^ In this study, network models of FC, SC, and PR all performed well in recognizing admission executive function/attention, implying that network features of functional and structural connections, as well as key brain regions all contribute to individual cognitive status of executive function/attention.

Referring to tests with two satisfactory ML classifiers, memory at admission can be recognized with FC and PR models, while postoperative aphasia can be predicted with SC and PR models. Notably, the PR models performed relatively better than connectivity in both tests, implying that connected regions have greater impact on the outcomes than regional connectivity. Additionally, language performance at admission can be recognized with the PR model, and the long‐term MES changes can be predicted with the FC model, indicating the significance of related regions and connections in each situation. Commonly, a brain network is composed of regions and their connections. Although clinical behaviors can be mapped within the brain connectome, based on our results, we suggest that each specific symptom or outcome is determined mainly by regions, connections, or both.^11^

Afterward, the SHAP method is performed in each effective model to acquire connectomic feature importance. In tests with at least two satisfactory models, components of SMN and DMN are noted in all models. The SMN belongs to the sensor cortex systems and is considered as the transducer to convert inputs into electrical signals and initiate physical responses.^16^ Conversely, the DMN is active in the resting state.^17^ Thus, these two networks are seemingly a good reflection of brain healthy. Because components of these two networks were among the top features in our results, we speculate that these two networks are good neuroimaging biomarkers for individual status recognition and treatment outcomes prediction.

The current study possesses several limitations or issues necessitating additional refinement or exploration. Primarily, we note that the top feature lists are different among models of the same test. For example, executive function/attention at admission was predicted by both FC and SC models; however, the most predictive features were connections between the right V7 and 5m, and between the right TPOJ3 and left MI, respectively. Such disparate regions and networks being predictive of the same function are prohibitive to intuitive interpretation. Further studies should be performed to disambiguate the functional and structural contributions to network effectivity. Second, while we analyze the largest sample of CSOV to date, and rely on fivefold cross validation, the sample size is still small from the perspective of ML models. Furthermore, the study lacks an independent test set. More data are necessary to achieve generalizable and stable individual classifiers of complicated clinical status. In addition, patients with larger cerebral infarctions are excluded as it becomes challenging to predict the outcomes of patients with only cerebral hypoperfusion. This exclusion criterion makes the study more beneficial for clinical decision‐making. Besides, large infarct lesions hamper the study of the internal neurons and their external FCs. Nevertheless, this study provides new insights into exploring clinical outcomes of CSOV from perspectives of individual brain connectome.

## 4 CONCLUSIONS

Our findings suggest that individual connectomic data derived from multimodal MRI is valuable for researches of brain ischemia with distortion. This study provides informative insights for how brain functions before and after reperfusion. Even in the context of experimental limitations, we are optimistic that the results will be integrated into public software in subsequent research and, by connecting to scanning devices, will offer clinical decision‐making recommendations for patients with cerebral ischemia.

## 5 MATERIALS AND METHODS

### 5.1 Participants

The study, which is observational in nature, received authorization from the ethics committee at Huashan Hospital (NO. 2014−278) and adhered to the principles outlined in the Declaration of Helsinki. Written informed consent was obtained from all participants. Inclusion criteria for patients included (1) right‐handed individuals of Chinese descent, aged from 18 to 70 years; (2) diagnosis of moyamoya disease, moyamoya syndrome, or cerebral occlusive disease determined by digital subtraction angiography^18^; (3) received extracranial–intracranial bypass surgery; (4) assessed by cognitive testing and multimodal MR scan at admission. Exclusion criteria for patients encompassed the presence of cortical or subcortical lesions exceeding 8 mm in their largest dimension on structural imaging,^19^ the existence of substantial neurological or psychiatric conditions, the presence of severe systemic illnesses or additional cerebrovascular disorders, or the use of specific medications like the benzodiazepine clonazepam.

We recruited patients from the general, north, and west campuses of our hospital, where they received care under consistent protocols and were operated on by skilled neurosurgeons at each respective campus. The surgical procedure was a combination of superficial temporal artery‐middle cerebral artery bypass and encephaloduromyosynangiosis.^20^ Bypass patency was confirmed through indocyanine green fluorescence imaging. Besides, the control group was selected from urban communities within Shanghai and underwent through MR angiography. Patients and controls were paired based on age, gender, level of education, and handedness.

### 5.2 Clinical assessment

A team of neurologists and neuropsychologists who were unaware of the patient diagnoses administered the assessment. Neurological status was evaluated using the NIHSS, an increase of which indicates unfavorable outcomes. Aphasia and motor paresis were common newly onset or aggravated symptoms after revascularizations due to either ischemic stroke or HPS.^5^, ^6^

Cognitive function was assessed through an extensive array of neuropsychological examinations, encompassing overall cognitive performance and four specific cognitive domains: memory, executive function/attention, language, and visuospatial function.^21^ For a general evaluation, the MES and Mini‐Mental State Examination (MMSE) were utilized, with higher scores signifying enhancement in condition. The diagnosis of domain impairment was made based on the criteria set forth by the AHA/ASA.^22^ For this research, impairment was defined as scoring 1.5 SDs away from the normative means of healthy controls on two tests within the same domain.

### 5.3 Multimodal MR image acquisition

Data were scanned on 3.0 Tesla MR systems. Referring to the general campus (Siemens Medical Solutions), the fMRI parameters encompassed gradient echo‐planar imaging, TR/TE of 2000/35 ms, FOV of 240 × 240 mm, and slice thickness of 4 mm. The structural images parameters encompassed a fast spoiled gradient recalled echo inversion recovery sequence, thick axial section of 1 mm, TR/TE of 1000/5 ms, FA of 20°, and FOV of 240 × 240 mm. The diffusion‐weighted images (DWI) parameters encompassed a slice thickness of 3 mm, FOV of 230 × 230 mm, diffusion‐weighted volumes of 20 with noncollinear directions (*b* = 1000 s/mm^2^) and nondiffusion‐weighted volumes of one (*b* = 0 s/mm^2^). Referring to the north campus (GE Healthcare), the fMRI parameters encompassed a TR/TE of 2000/30 ms, FOV of 220 × 220 mm, and slice thickness of 3.2 mm. The structural images parameters encompassed a thick axial section of 1 mm, TR/TE of 8100/3.2 ms, FA of 12°, and FOV of 256 × 256 mm. The DWI parameters encompassed a slice thickness of 1.5 mm, FOV of 270 × 270 mm, diffusion‐weighted volumes of 30 with noncollinear directions (*b* = 1000 s/mm^2^) and nondiffusion‐weighted volumes of five (*b* = 0 s/mm^2^). Referring to the west campus (Philip Medical Systems), the fMRI parameters encompassed a TR/TE of 2000/35 ms, FOV of 210 × 210 mm, and slice thickness of 4 mm. The structural images parameters encompassed a thick axial section of 1 mm, shortest TR/TE, FA of 8°, and FOV of 240 × 240 mm. The DWI parameters encompassed a slice thickness of 5 mm, FOV of 224 × 224 mm, diffusion‐weighted volumes of 15 with noncollinear directions (*b* = 1000 s/mm^2^) and nondiffusion‐weighted volumes of one (*b* = 0 s/mm^2^).

### 5.4 Data preprocessing

#### 5.4.1 Diffusion tractography preprocessing

The DWI underwent processing through the Infinitome software from Omniscient Neurotechnology (https://o8t.com), which used standard procedures written with the Python language.^23^ The specific procedures have been outlined in another published articles of our collaborative team.^14^

#### 5.4.2 Developing an individualized brain atlas through ML‐driven parcellation

To reduce the impact of individual gyrus differences, a ML‐driven, personalized adaptation of the Human Connectome Project Multimodal Parcellation 1.0 (HCP‐MMP1) atlas was employed.^13^ Briefly, the process involved training a ML algorithm on a distinct group of 200 healthy controls, initially by processing their DWI and T1 scans as previously described. Subsequently, a NIFTI MNI space version of the HCP‐MMP1 atlas was morphed to fit each individual brain, and SC metrics were computed for every atlas pair and a predefined set of regions of interest (ROIs). These ROIs encompassed eight subcortical structures in each hemisphere, alongside the brainstem, identified by the streamlines that concluded within an ROI. This phase facilitates the creation of feature vectors and the generation of a parcellation centroid. This centroid is then used to limit the voxels examined, with the aim to assign them to a specific parcellation that is within a reasonable proximity of its standard location. The feature vectors corresponding to each region were subsequently utilized as a training dataset, and the data were modeled employing the XGBoost technique.^24^

This developed model was then deployed on a new subject by initially morphing the HCP‐MMP1 atlas to fit the new brain, followed by the extraction of a set of feature vectors for the connectivity of each voxel. Subsequently, these feature vectors were employed to ascertain whether each voxel was part of a specific parcellation region, and if it was determined to be so, the voxel was allocated to that particular parcellation. In this way, a version of the HCP‐MMP1 atlas was constructed, comprising 180 cortical regions and nine subcortical structures per hemisphere, as well as a single brainstem region. This version was tailored to each subject and remained unaffected by variations in brain shape or pathological distortions. Furthermore, the parcels that were detected were automatically categorized by the Infinitome software according to their recognized associations with large‐scale brain networks (Figure S1).

#### 5.4.3 Resting‐state FMRI preprocessing

The processing of the data entailed a series of essential steps, which included: (1) stabilizing the T1 and BOLD images by performing motion correction through rigid body transformations; (2) removing any image slices that showed significant movement artifacts; (3) utilizing a CNN for skull stripping on T1 images, which were then inverted, rigidly matched to the BOLD data, and subsequently utilized to facilitate skull removal from the fMRI images; (4) rectifying timing discrepancies between slices with slice timing correction and equalizing the signal levels across all images using global intensity normalization; (5) applying a diffeomorphic mapping approach to correct for gradient field distortions, thereby enhancing the local alignment between the fMRI and T1 images; (6) high‐variance nuisance variables were calculated using the CompCor technique.^25^ These variables, together with motion‐related confounds, were subsequently removed from the fMRI dataset. The procedure also entailed the detrending of the fMRI time series by eliminating linear and quadratic components, and it is important to highlight that this method abstained from conducting global signal regression; (7) applying a smoothing filter with a FWHM of 4 mm. The tailored atlas developed in earlier stages was mapped onto the T1 image and targeted to the regions of grey matter. This positioning was ideal for the retrieval of an average BOLD signal time series across all 379 designated areas (180 parcellations × 2 hemispheres, plus 19 subcortical structures), and resulted in a total of 143,641 correlational values.

Several techniques were used to minimize the impact of vendor specific variabilities. These include: (1) resampling fMRI and DWI voxel grids to a standard 2 mm voxel size across all scans. (2) The fMRI preprocessing (slice time correction, motion correction) to reduce site specific impacts. (3) Registration based distortion correction of both fMRI and DWI to reduce site specific gradient distortions. (4) High variance nuisance regression via the CompCor algorithm also helps standardize the signal for comparison across subjects.

#### 5.4.4 ML classification and feature extraction

The ML techniques were employed to predict the performance on tests for everyone, drawing on the pairwise FC, the SC among the 379 areas of the personalized brain atlas, or the PR metrics for each region, thereby creating a trio of predictive models for every test administered. For every model constructed, variables including age, gender, and surgical side were factored in as predictors, and the analysis was conducted using the XGBoost Classification algorithm. A fivefold cross‐validation approach was employed to assess each model, and performance was measured using the average area under the receiver operating characteristic curve (AUC–ROC) ± standard deviation. An AUC score exceeding 0.7 indicated that the model could yield reliable classification results. A total of 11 tests were trained, including (1) the neurological (NIHSS_pre_ scores) and cognitive status (four cognitive domains) at admission; (2) perioperative outcomes of general status (NIHSS_peri_–NIHSS_pre_ scores) and specific newly onset or aggravated complications (aphasia and motor paresis); (3) long‐term outcomes of the neurological (NIHSS_LTFU_–NIHSS_pre_ scores) and cognitive status (MMSE_LTFU_–MMSE_pre_ scores and MES_LTFU_–MES_pre_ scores) (Table S1). Tests with at least two good models are deemed effective.

Extracting features from ML models utilizing large datasets is difficult, especially when investigating the magnitude and directionality of the features. This is however necessary given the need‐to‐know which parts of the brain are associated with pathology in clinical practice. For each model, we produced a representation of the importance of each large‐scale brain network in the output of the model, and a SHAP plot of the top 20 features contributing to the model. The SHAP method calculates feature importance by deriving Shapley values for each feature.^26^ Each SHAP plot provides a list of features in descending order of importance, along with their impact on the model on the x‐axis, with the color of each point, representing a single observation, indicating whether a high (red) or low (blue) value of that feature is associated with the classification. The schematic description of the conceptual basis behind the data processing is shown in Figure S2.

## AUTHOR CONTRIBUTIONS

Yu Lei and Xin Zhang wrote the original draft; Ying Mao and Michael E. Sughrue served as scientific advisors. Yuxiang Gu critically reviewed the study proposal. Wei Ni and Chao Gao participated in the surgery. Heng Yang, Yanjiang Li, and Xinjie Gao cared for study patients and arranged the follow‐ups. Ding Xia and Xia Zhang collected data. Karol Osipowicz and Stephane Doyen performed the data processing. All authors have read and approved the final manuscript.

## CONFLICT OF INTEREST STATEMENT

Karol Osipowicz, Stephane Doyen, and Michael E. Sughrue are employees in Omniscient Neurotechnology, but have no potential relevant financial or nonfinancial interests to disclose. The other authors declare that they have no conflict of interests.

## ETHICS STATEMENT

The study protocol was approved by the Ethics Committee of Huashan Hospital (NO. 2014−278). Written informed consent was obtained from all participants.

## Supporting information

Supporting Information

## Data Availability

The data supporting the findings of this study are available upon reasonable request from the corresponding author following publication.
