# Supplementary material for: Application of individual brain connectome in chronic ischemia: mapping symptoms before and after reperfusion
Source: MedComm (2020). 2024 Jun 2;5(6):e585. doi: 10.1002/mco2.585 (PMC11144839; doi:10.1002/mco2.585)
Supplement: Supplementary file 1 — Supporting Information [file MCO2-5-e585-s001.docx]

**Application of Individual Brain Connectome in Chronic Ischemia: Mapping Symptoms Before and After Reperfusion**

**Short Title**: Individual brain connectome in ischemia

Yu Lei^1,2,3,4,5#^, Xin Zhang^1,2,3,4,5#^, Wei Ni^1,2,3,4,5^, Chao Gao^1,2,3,4,5^, Yanjiang Li^1,2,3,4,5^, Heng Yang^1,2,3,4,5^, Xinjie Gao^1,2,3,4,5^, Ding Xia^6^, Xia Zhang^7^, Karol Osipowicz^8^, Stephane Doyen^8^, Michael E. Sughrue^7,8^, Yuxiang Gu^1,2,3,4,5*^, and Ying Mao^1,2,3,4,5*^

^1^ Department of Neurosurgery, Huashan Hospital, Fudan University, Shanghai, China

^2^ National Center for Neurological Disorders, Shanghai, China

^3^ Shanghai Key Laboratory of Brain Function and Restoration and Neural Regeneration, Shanghai, China

^4^ Neurosurgical Institute of Fudan University, Shanghai, China

^5^ Shanghai Clinical Medical Center of Neurosurgery, Shanghai, China

^6^ Department of Radiology, Huashan Hospital, Fudan University, Shanghai, China

^7^ International Joint Research Center on Precision Brain Medicine, XD Group Hospital, Xi’an, China

^8^ Omniscient Neurotechnology, Sydney, New South Wales, Australia

***Correspondence should be addressed to:** Yuxiang Gu (Email: guyuxiang1972@126.com), Department of Neurosurgery, Huashan Hospital, Fudan University, Shanghai, China; Ying Mao (Email: maoying@fudan.edu.cn), Huashan Hospital, Fudan University, Shanghai, China. ^#^These authors contributed equally to this work.

**Table S1** Binarization applied to each machine learning test.

| **Target** | **Binarization** |
| --- | --- |
| *Admission status* | |
| NIHSS score | Class 0: 0  Class 1: > 0 |
| Memory | Class 0: Defective  Class 1: Intact |
| Executive function/attention | Class 0: Defective  Class 1: Intact |
| Language | Class 0: Defective  Class 1: Intact |
| Visuospatial function | Class 0: Defective  Class 1: Intact |
| *Short-term outcomes* | |
| NIHSS changes | Class 0: NIHSS_peri_ - NIHSS_pre_ ≤ 0  Class 1: NIHSS_peri_ - NIHSS_pre_ > 0 |
| Aphasia | Class 0: newly-onset or aggravated  Class 1: same as admission |
| Motor paresis | Class 0: newly-onset or aggravated  Class 1: same as admission |
| *Long-term outcomes* | |
| NIHSS changes | Class 0: NIHSS_LTFU_ - NIHSS_pre_ ≤ 0  Class 1: NIHSS_LTFU_ - NIHSS_pre_ > 0 |
| MMSE changes | Class 0: MMSE_LTFU_ - MMSE_pre_ > 0  Class 1: MMSE_LTFU_ - MMSE_pre_ ≤ 0 |
| MES changes | Class 0: MES_LTFU_ - MES_pre_ > 0  Class 1: MES_LTFU_ - MES_pre_ ≤ 0 |
| Gender | Class 0: male  Class 1: female |

**Table S2** Abbreviation list of brain regions in the main text.

| **Abbr.** | **Regions** | **Abbr.** | **Regions** |
| --- | --- | --- | --- |
| A4 | Auditory area 4 of auditory association cortex | A5 | Auditory area 5 of auditory network |
| 5mv | Area of posterior cingulate cortex | 47s | Part of lateral frontal lobe |
| IFSa | Inferior frontal sulcus anterior area in dorsolateral frontal lobe | MI | Primary motor cortex |
| MT | Middle temporal area | POS1 | Parietal operculum 1 in the medial parietal lobe |
| 6mp | Area of supplemental motor area | STSda | Superior temporal sulcus dorsal anterior |
| STV | Superior temporal visual area | 10r | 10 rostral, area of anterior cingulate cortex |
| TPOJ3 | Temporal-parieto-occipital junction 3 | ventralDC | Ventral diencephalon |
| V7 | Area of dorsal visual stream |  |  |

**Supplementary Figures**


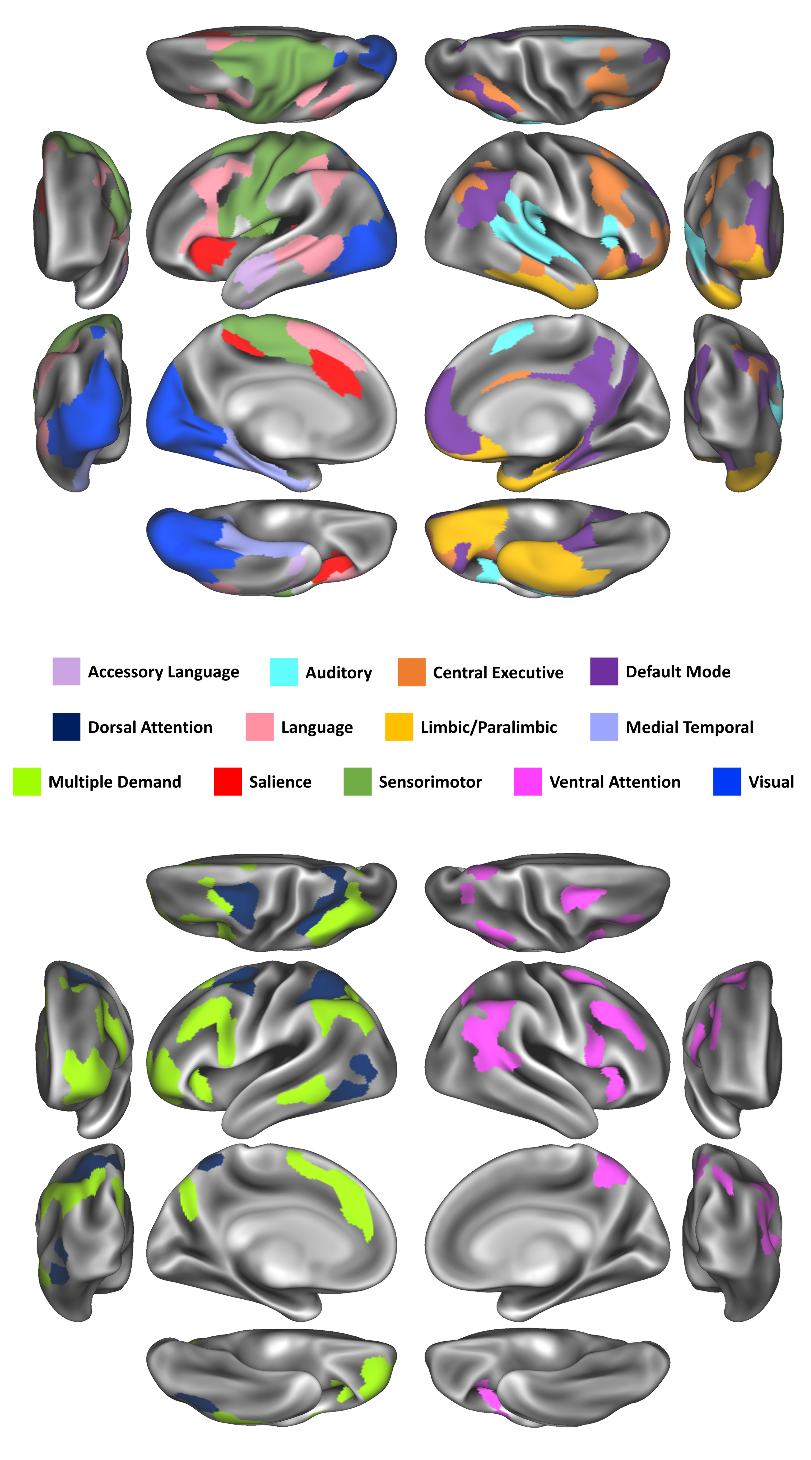


**Figure S1.** A visual representation of the large-scale networks, which includes the auditory, accessory language, central executive, default mode, dorsal attention, language, limbic/paralimbic, multiple demand, salience, sensorimotor, ventral attention, and visual networks, as well as the medial temporal region. The multiple demand, dorsal attention, and ventral attention networks have been shown separately since some of their components overlap with other networks.


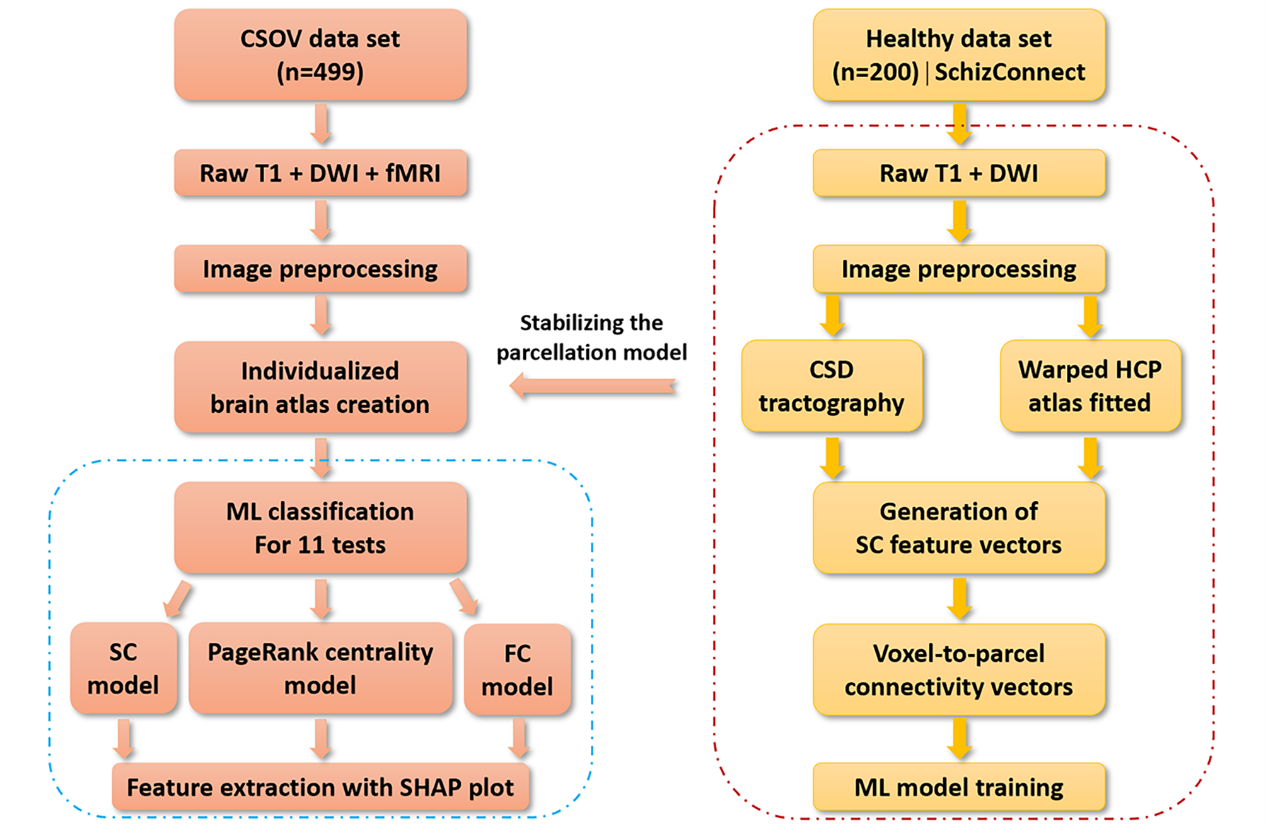


**Figure S2.** Schematic description of the multi-modal data preprocessing to achieve individual brain connectome, and the deep-learning models construction and features extraction from individual structural connectivity, functional connectivity, and PageRank centrality.


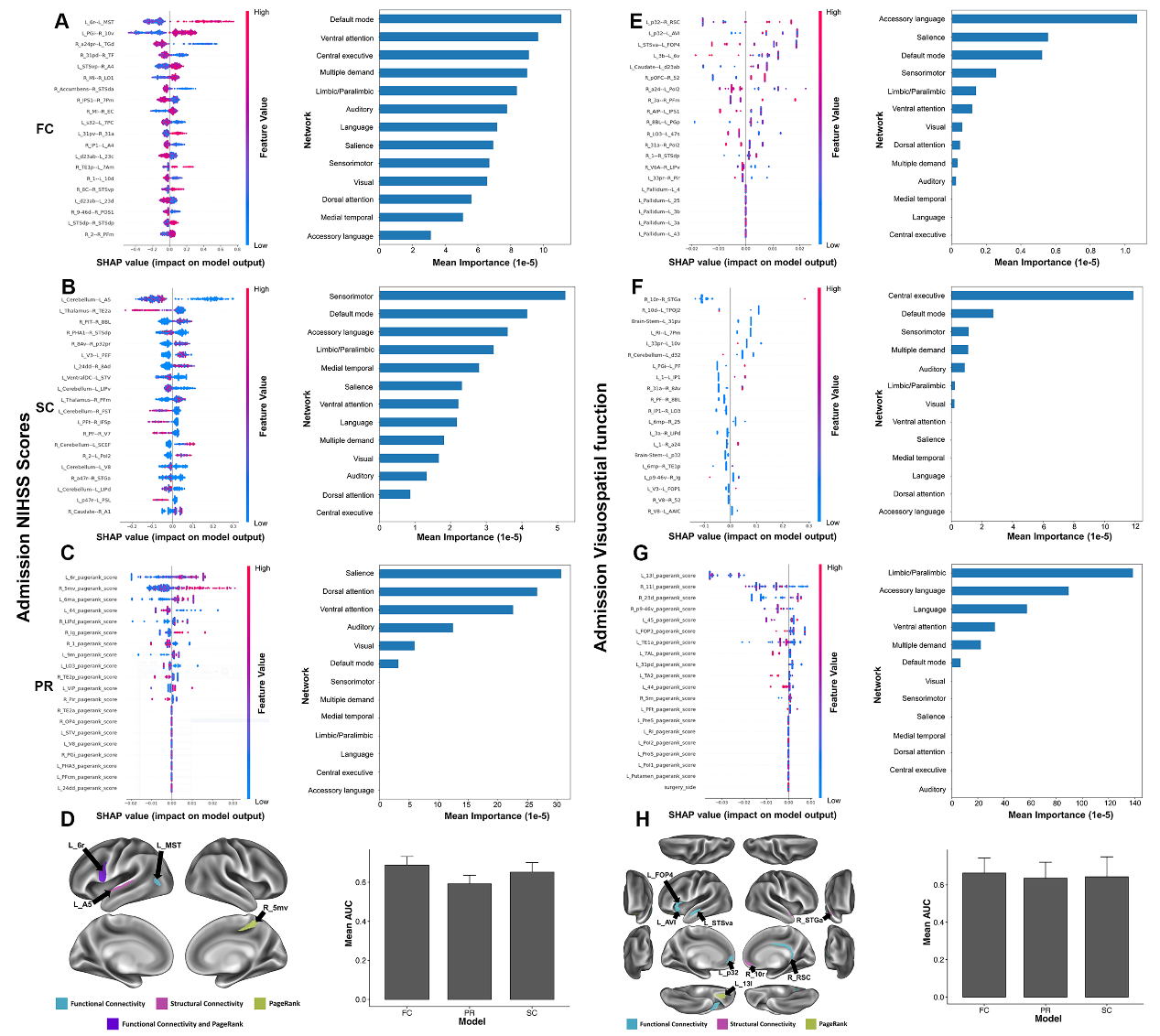


**Figure S3.** The SHAP plot of feature importance for models classifying admission NIHSS score (A-C) and visuospatial function (E-G) at the single-subject level. Top areal features of brain connectome from each model are represented on a model brain (D,H). The left Cerebellum is also a top feature in the SC model of NIHSS score.


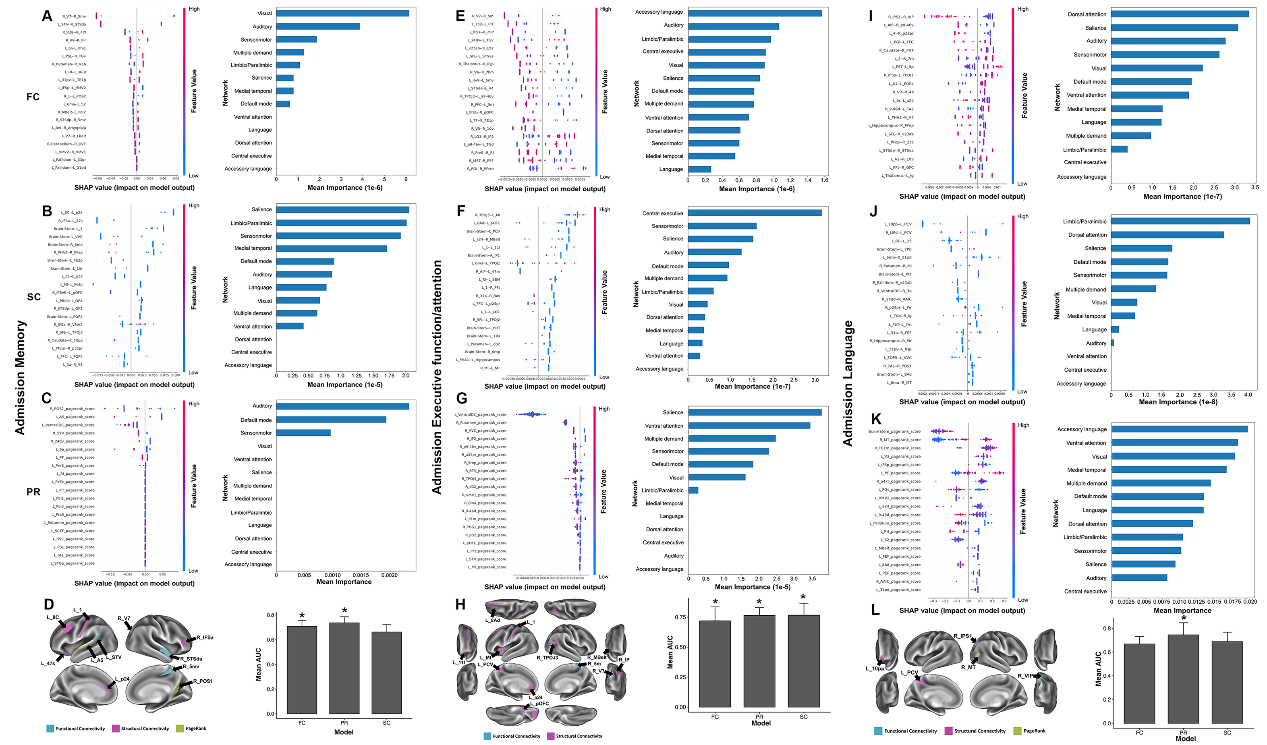


**Figure S4.** The SHAP plot of feature importance for models classifying tests of admission memory (A-C), executive function/attention (E-G), and language (I-K) at the single-subject level. Note that the brainstem, and left ventral diencephalon are also top features of the SC and PR models in testing executive function/attention, respectively. And the brainstem is also a top feature in the PR model in testing language. Top areal features of brain connectome from each model are represented on a model brain (D, H, L). One asterisk means good performance of a model.


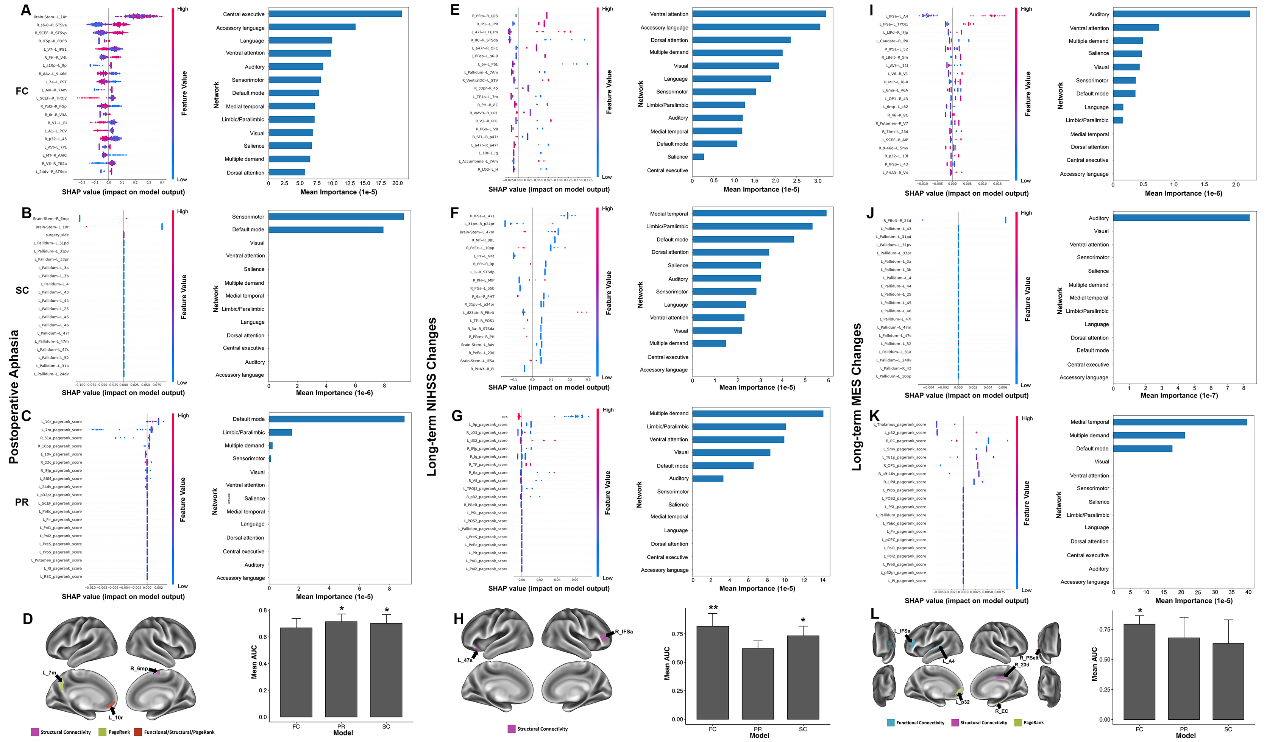


**Figure S5.** The SHAP plot of feature importance for models classifying tests of postoperative aphasia (A-C), long-term NIHSS changes (E-G), and long-term MES changes (I-K) at the single-subject level. Note that the brainstem is also a top feature in the SC model in testing both postoperative aphasia and long-term NIHSS changes. Top areal features of brain connectome from each model are represented on a model brain **(**D, H, L**)**. One asterisk means good performance of a model. Two asterisks mean very good performance.


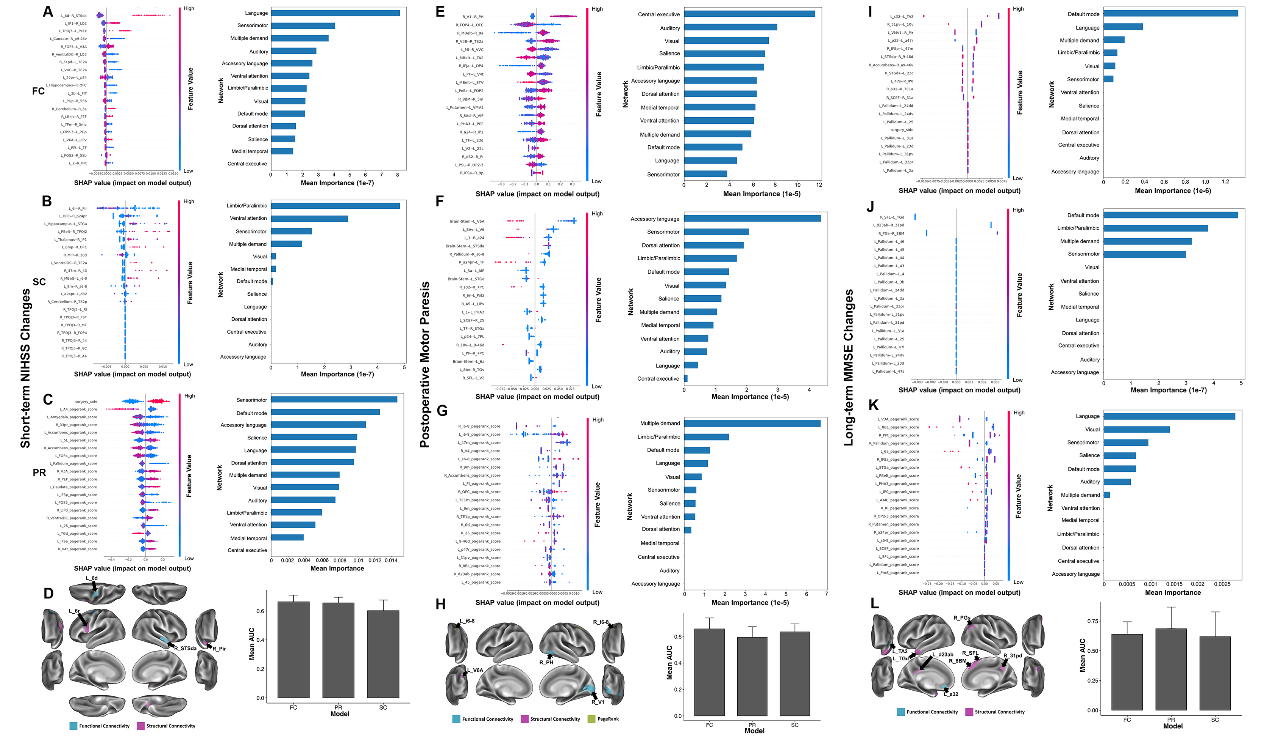


**Figure S6.** The SHAP plot of feature importance for models classifying short-term NIHSS changes (A-C), postoperative motor paresis (E-G), and long-term MMSE changes (I-K) at the single-subject level. Top areal features of brain connectome from each model are represented on a model brain (D,H,L).

**
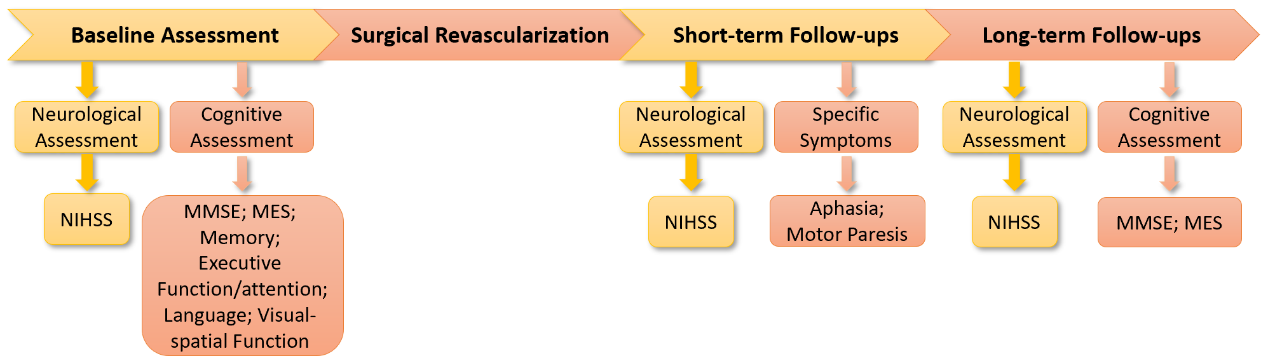
**

**Figure S7.** The timeline for the evaluation of the prospective cohort study.

**Detailed analysis of ML models with poor performance (AUC<0.7).**

*1.1 Admission Neurological Status*

Referring to the key features of individual brain connectome to recognize admission NIHSS scores, a high FC between the left 6r and left MST is the top feature, while the default mode network has the highest mean importance at the network level (Suppl Fig.2a). Next, a low SC between the left cerebellum and left A5 stands out, while at the network level, the sensorimotor network has the highest contribution (Suppl Fig.2b). Finally, a high PR score of the left 6r and right 5mv are the top features, while the salience network has the highest mean importance (Suppl Fig.2c).

*1.2 Admission Cognitive Status*

In SHAP analysis of memory, a low SC between the left 8C and left p24 is the top feature, while the salience network has the highest mean importance at the network level (Fig.2b).

Referring to the language, a high FC between the right IPS1 and right VIP is the top feature. Among the networks, the dorsal attention network is the top feature (Fig.2i). Besides, a high SC between the left 10pp and left PCV is the top feature, while the rest of the features overlap in their contribution. The limbic/paralimbic network is the top feature at the network level (Fig.2j).

Referring to the visuospatial function, a high FC between the left p32 and right RSC is the top feature, though there is significant overlap among features. Among the networks, the accessory language network is the top feature (Suppl Fig.2e). Next, a high SC between right 10r and right STGa is the top feature, showing an association with intact visuospatial function, while a low SC is associated with dysfunction. The central executive network is the top feature at the network level (Suppl Fig.2f). Finally, the PR score of the left 13l is the top feature, and the limbic/paralimbic network contribute most at the network level (Suppl Fig.2g).

*2.1 Short-term Neurological Outcomes*

In SHAP analysis of NIHSS changes, the high FC between left 6d and right STSda is the top feature, and the language network has the highest importance at the network level (Suppl Fig.3a). Next, the low SC between left 6r and right Pir is the top feature, but there is too much variation among the features. Among the network, the limbic/paralimbic network has the greatest contribution (Suppl Fig.3b). Referring to the PR score, there is a significant degree of overlap and variability among the contributions of features, though the surgery side is ranked first. The sensorimotor network is the top feature at the network level (Suppl Fig.3c)

*2.2 Specific Newly-onset Or Aggravated Complications*

Referring to the post-operative aphasia, a high FC between the brainstem and left 10r is the top feature, though there is high variability among the individual observations. Among the networks, the central executive network contributes most (Fig.3a).

In SHAP analysis of post-operative motor paresis, a high FC between right V1 and right PH is the top feature, while the central executive network has the highest mean importance at the network level (Suppl Fig.3e). Next, a low SC between the brainstem and left V6A, and the accessory language network at the network level have the greatest contribution (Suppl Fig.3f). Finally, the PR scores of the right and left i6-8 are the top two features (Suppl Fig.3g). For the right i6-8, individual feature values are mixed for low SHAP value predictions. For the left i6-8, low individual feature values predict low SHAP scores, while high scores are predicted by a mix of feature values. At the network level, the multiple demand network is the top feature (Suppl Fig.3g).

*3.1 Long-term Neurological Outcomes*

In SHAP analysis of NIHSS changes, sex has the highest feature importance with PR score, while the multiple demand network has the greatest contribution at the network level (Fig.3g).

*3.2 Long-term Cognitive Outcomes*

Referring to the MMSE changes, the FC and SC models both show low variance in individual feature values, thus the direction of any effect should be interpreted with caution. The FC between left s32 and left TA2 has the highest feature importance. Among the networks, the default mode network is the top feature (Suppl Fig.3i). Next, the low SC between the right SFL and left TGd is the top feature, while the default mode network is the top feature at the network level (Suppl Fig.3j). In addition, no feature stands out at the individual region level based on PR score, but the language network is the top feature at the network level (Suppl Fig.3k).

In SHAP analysis of MES changes, the low SC between the right PBelt and right 23d is the top feature, while the auditory network stands out at the network level (Fig.3j). Besides, the PR score of the left thalamus is the top feature, while the medial temporal region is the top feature at the network level (Fig.3k).
